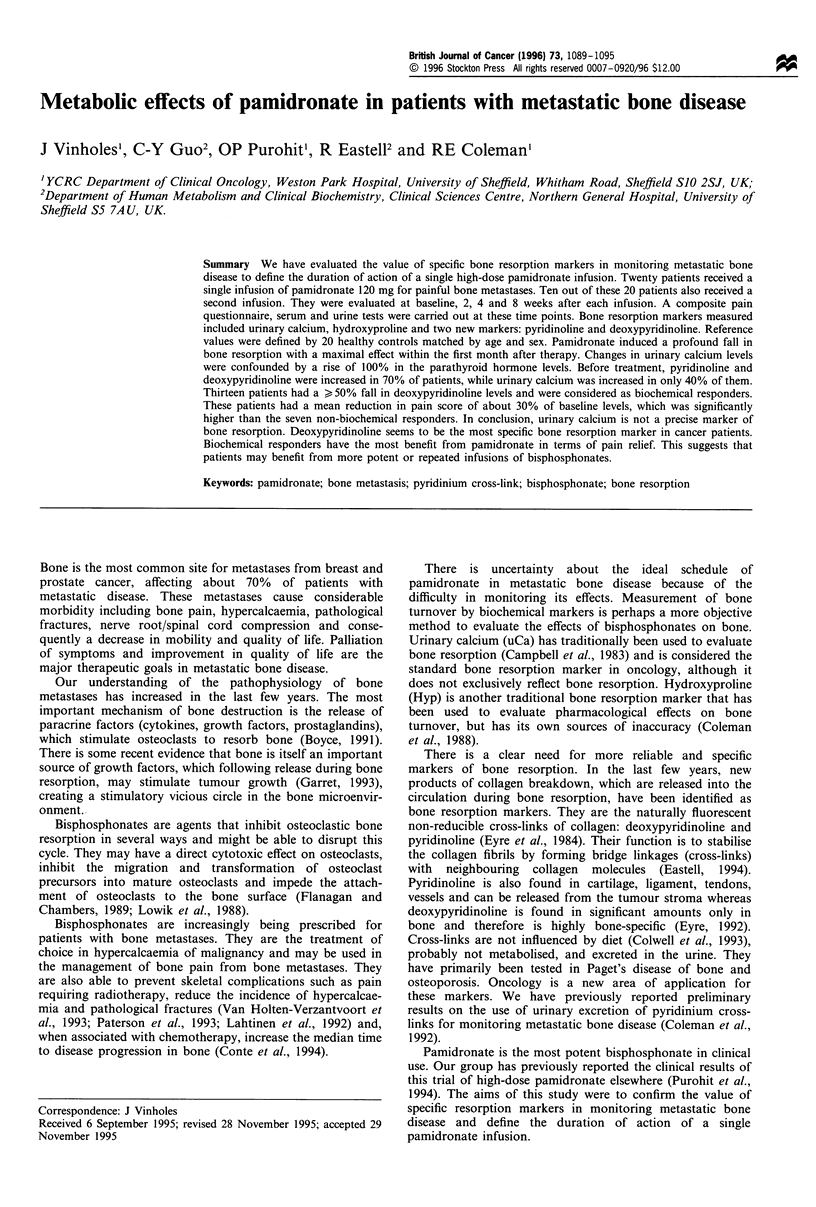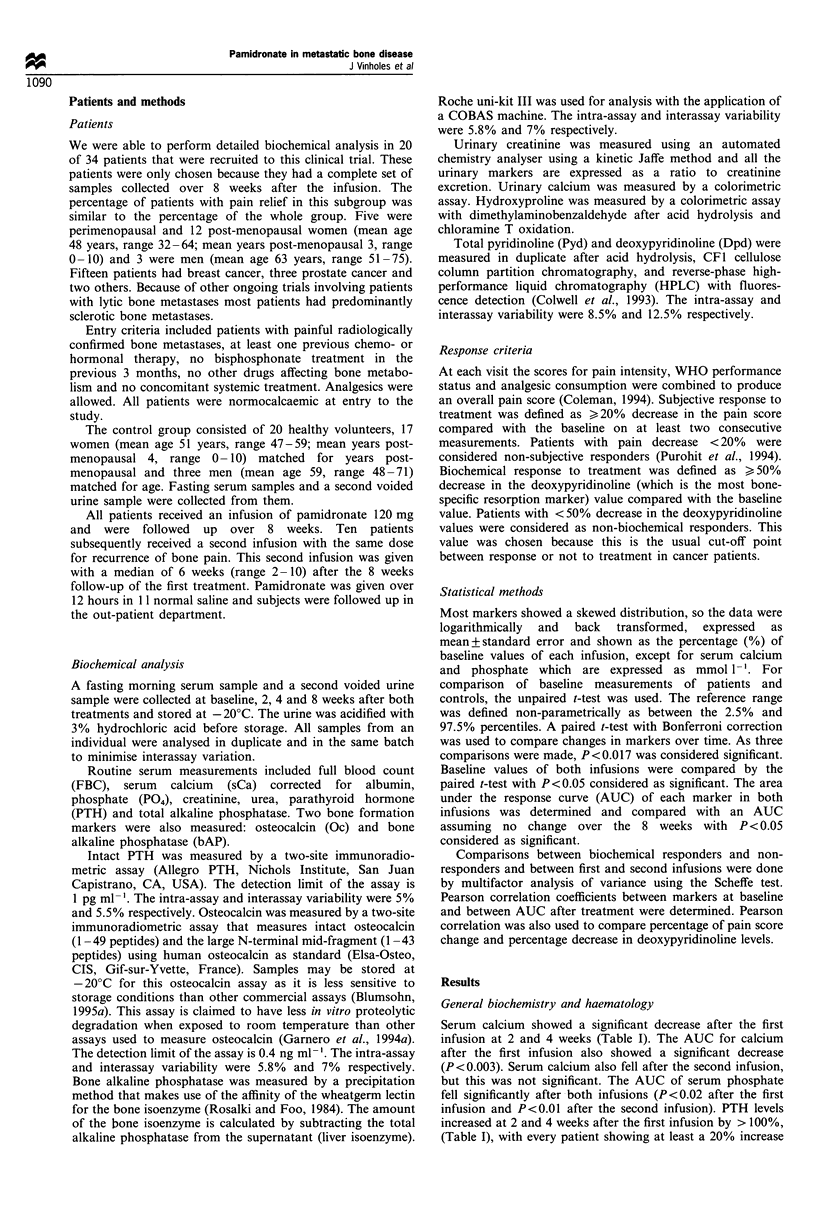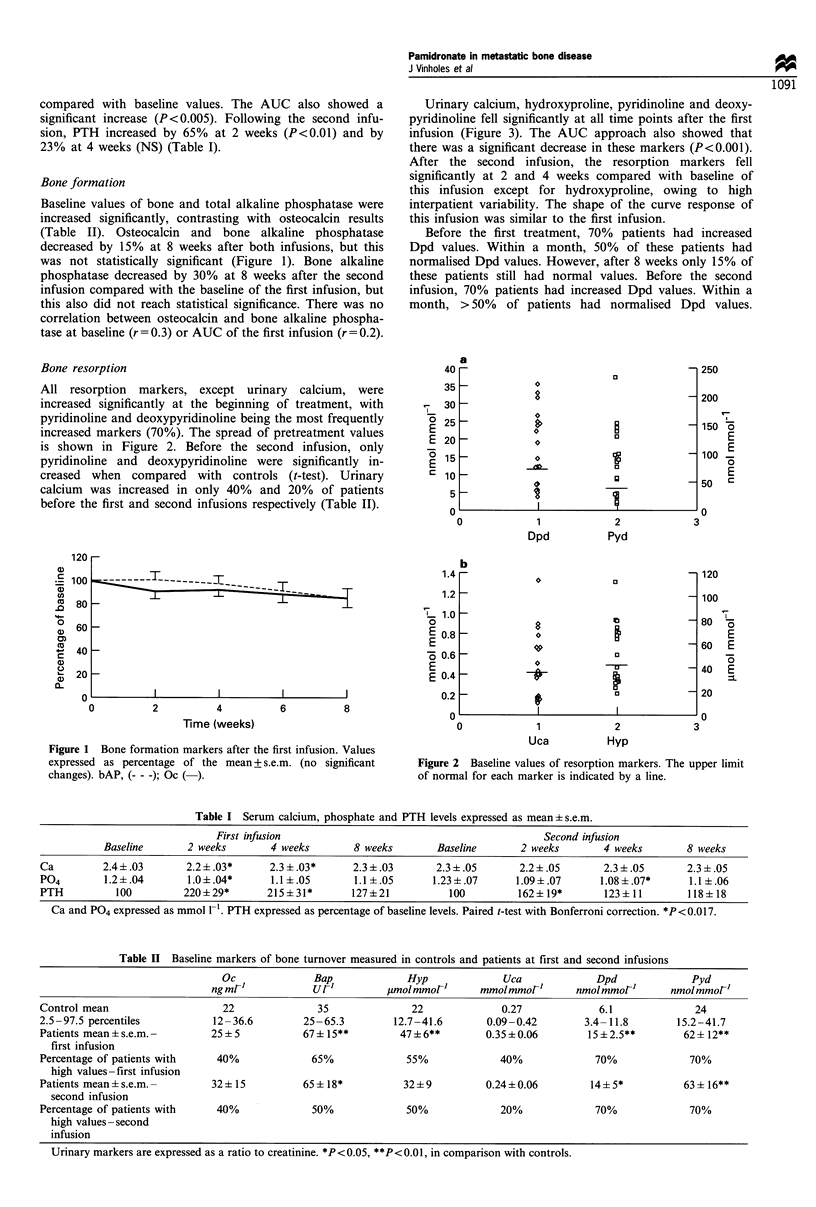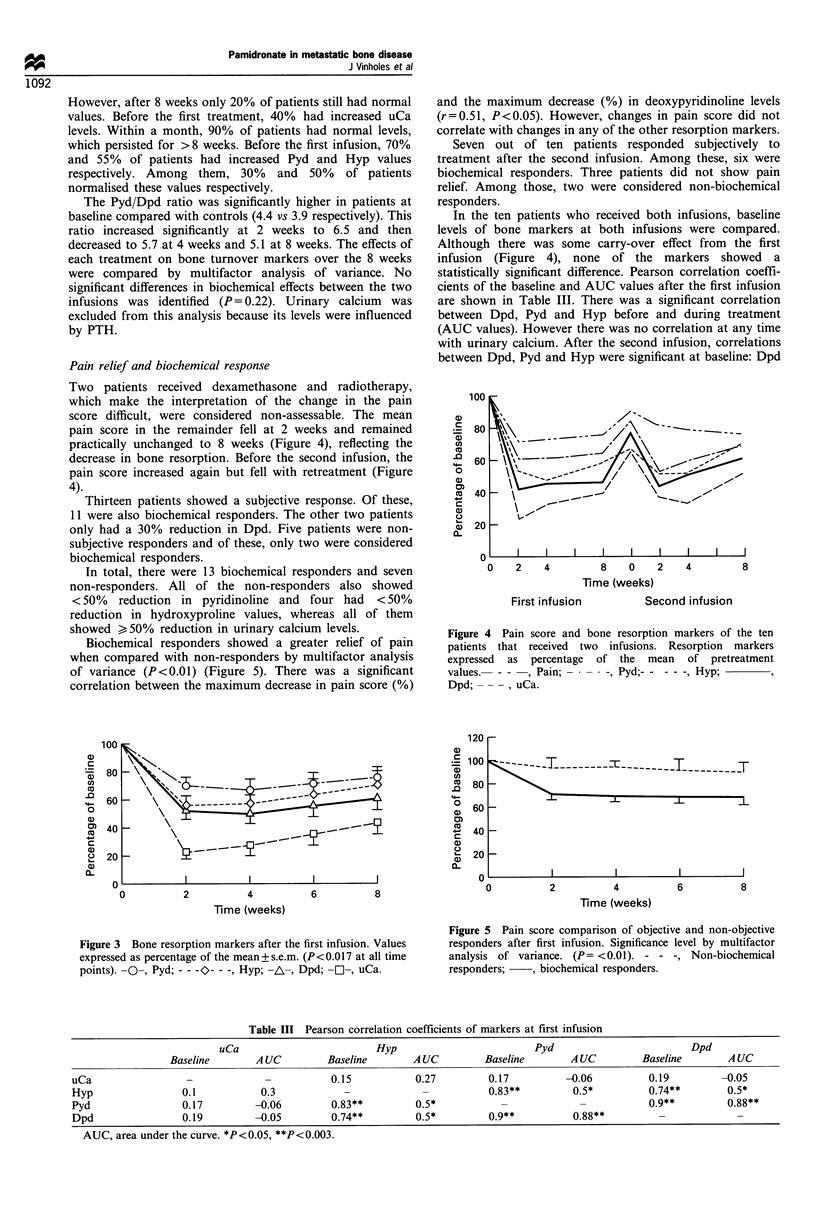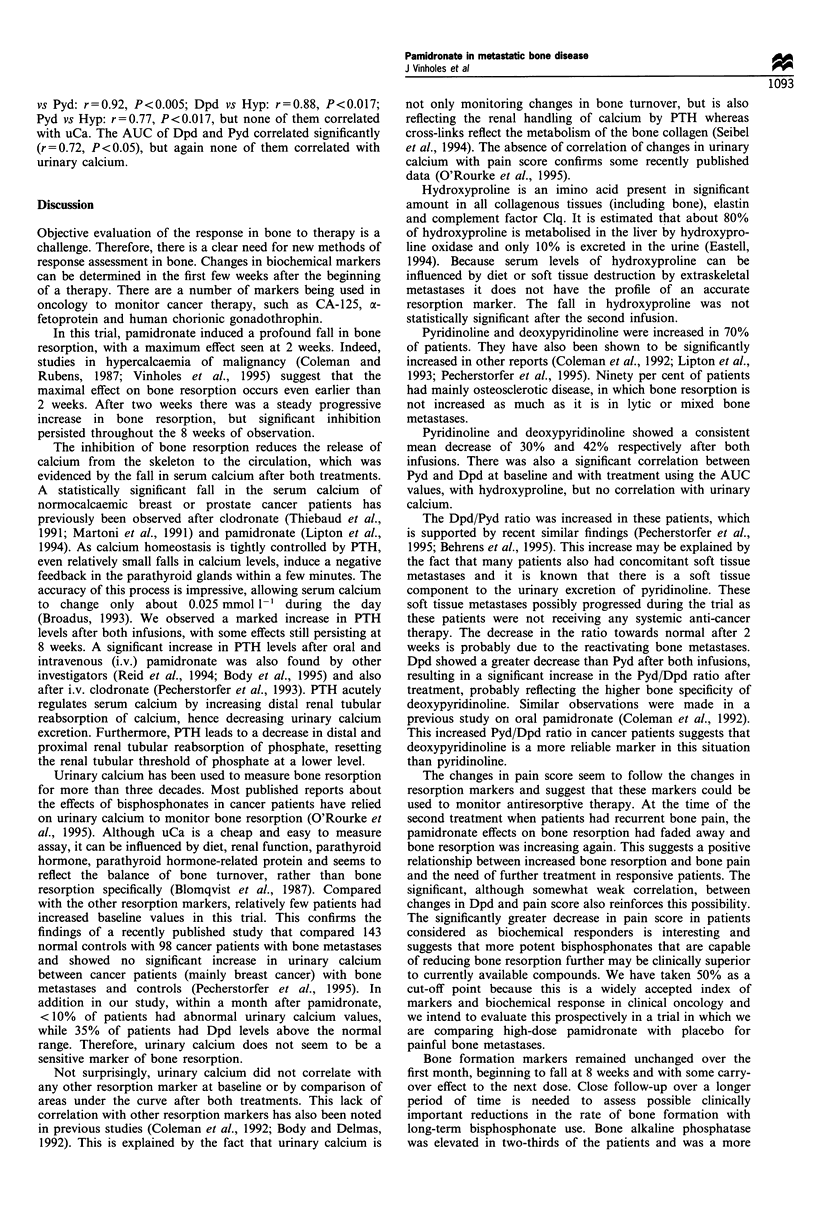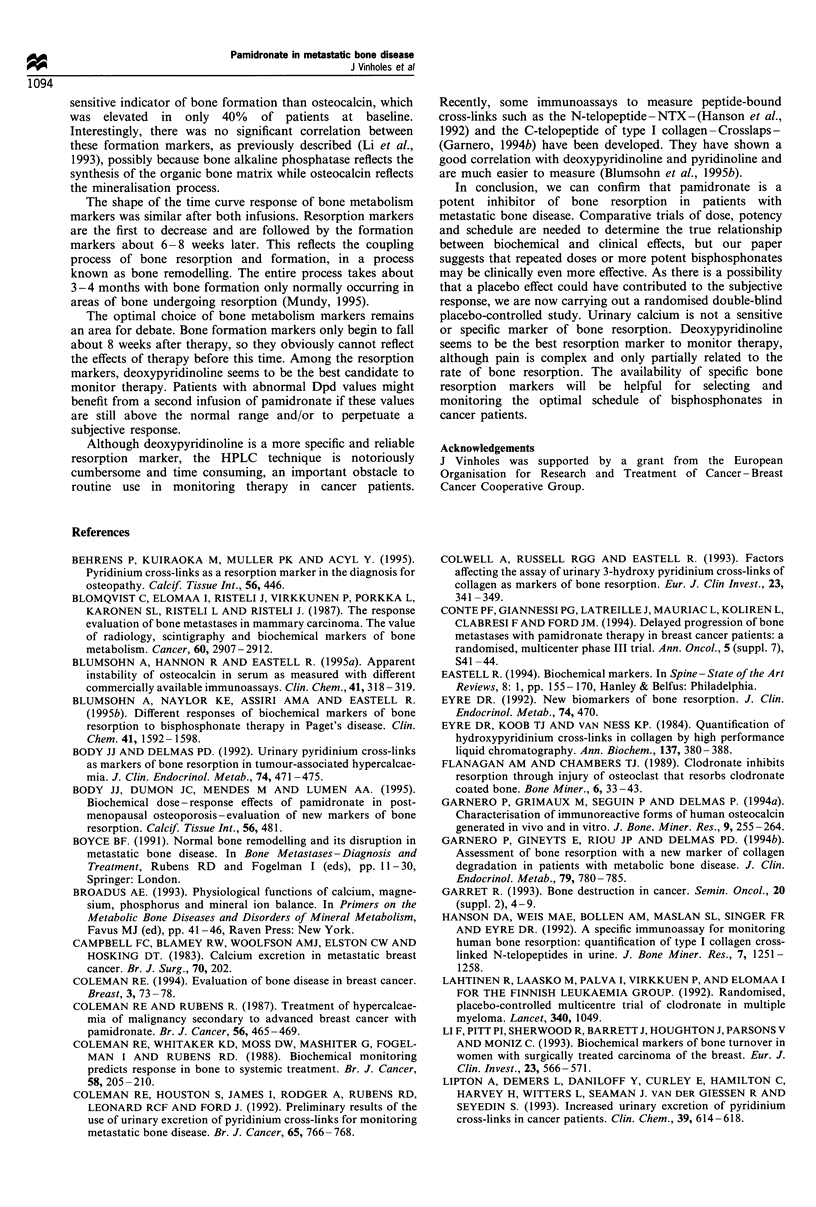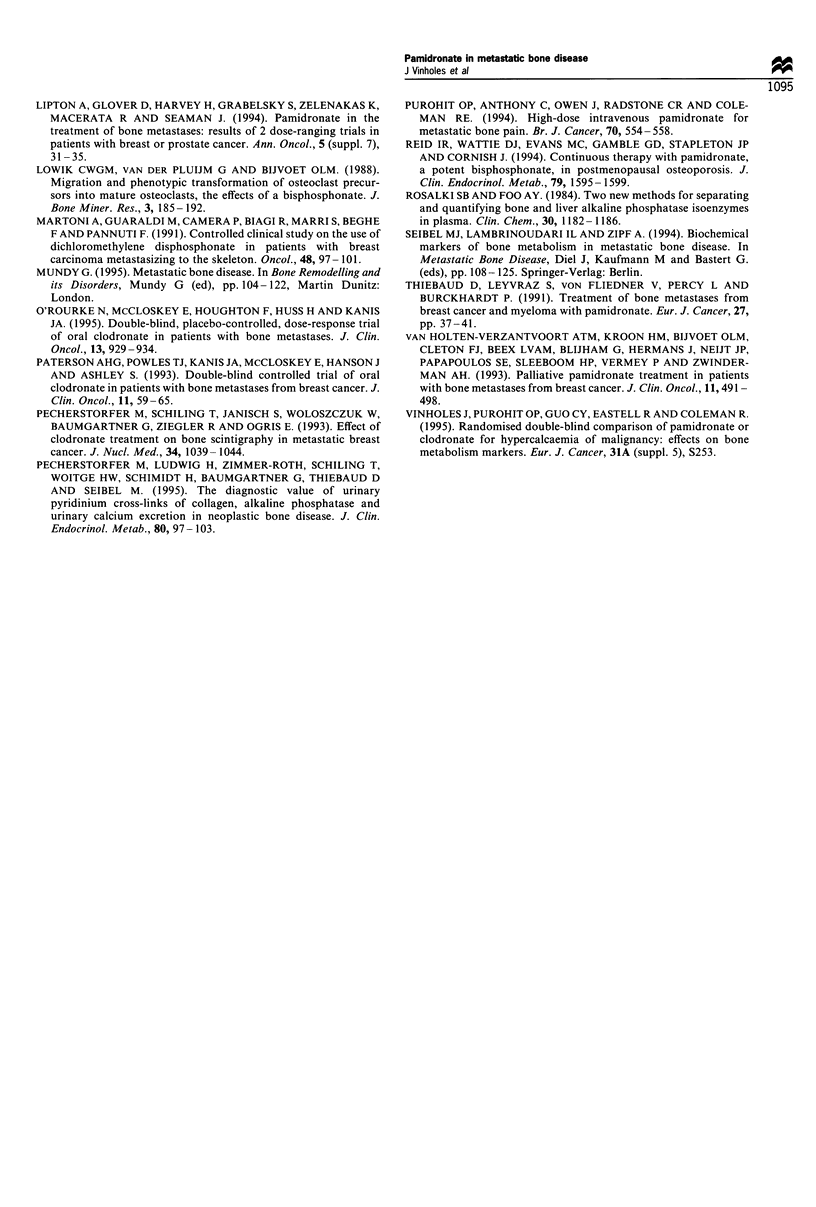# Metabolic effects of pamidronate in patients with metastatic bone disease.

**DOI:** 10.1038/bjc.1996.210

**Published:** 1996-05

**Authors:** J. Vinholes, C. Y. Guo, O. P. Purohit, R. Eastell, R. E. Coleman

**Affiliations:** YCRC Department of Clinical Oncology, Weston Park Hospital, University of Sheffield, UK.

## Abstract

We have evaluated the value of specific bone resorption markers in monitoring metastatic bone disease to define the duration of action of a single high-dose pamidronate infusion. Twenty patients received a single infusion of pamidronate 120 mg for painful bone metastases. Ten out of these 20 patients also received a second infusion. They were evaluated at baseline, 2, 4 and 8 weeks after each infusion. A composite pain questionnaire, serum and urine tests were carried out at these time points. Bone resorption markers measured included urinary calcium, hydroxyproline and two new markers: pyridinoline and deoxypyridinoline. Reference values were defined by 20 healthy controls matched by age and sex. Pamidronate induced a profound fall in bone resorption with a maximal effect within the first month after therapy. Changes in urinary calcium levels were confounded by a rise of 100% in the parathyroid hormone levels. Before treatment, pyridinoline and deoxypyridinoline were increased in 70% of patients, while urinary calcium was increased in only 40% of them. Thirteen patients had a > or = 50% fall in deoxypyridinoline levels and were considered as biochemical responders. These patients had a mean reduction in pain score of about 30% of baseline levels, which was significantly higher than the seven non-biochemical responders. In conclusion, urinary calcium is not a precise marker of bone resorption. Deoxypyridinoline seems to be the most specific bone resorption marker in cancer patients. Biochemical responders have the most benefit from pamidronate in terms of pain relief. This suggests that patients may benefit from more potent or repeated infusions of bisphosphonates.


British Journal of Cancer (1996) 73, 1089–1095
© 1996 Stockton Press All rights reserved 0007–0920/96 $12.00

### Metabolic effects of pamidronate in patients with metastatic bone disease

J Vinholes[1], C-Y Guo[2], OP Purohit[1], R Eastell[2] and RE Coleman[1]

[1]*YCRC Department of Clinical Oncology, Weston Park Hospital, University of Sheffield, Whitham Road, Sheffield S10 2SJ, UK;* [2]*Department of Human Metabolism and Clinical Biochemistry, Clinical Sciences Centre, Northern General Hospital, University of Sheffield S5 7AU, UK.*

**Summary** We have evaluated the value of specific bone resorption markers in monitoring metastatic bone disease to define the duration of action of a single high-dose pamidronate infusion. Twenty patients received a single infusion of pamidronate 120 mg for painful bone metastases. Ten out of these 20 patients also received a second infusion. They were evaluated at baseline, 2, 4 and 8 weeks after each infusion. A composite pain questionnaire, serum and urine tests were carried out at these time points. Bone resorption markers measured included urinary calcium, hydroxyproline and two new markers: pyridinoline and deoxypyridinoline. Reference values were defined by 20 healthy controls matched by age and sex. Pamidronate induced a profound fall in bone resorption with a maximal effect within the first month after therapy. Changes in urinary calcium levels were confounded by a rise of 100% in the parathyroid hormone levels. Before treatment, pyridinoline and deoxypyridinoline were increased in 70% of patients, while urinary calcium was increased in only 40% of them. Thirteen patients had a $\geqslant$50% fall in deoxypyridinoline levels and were considered as biochemical responders. These patients had a mean reduction in pain score of about 30% of baseline levels, which was significantly higher than the seven non-biochemical responders. In conclusion, urinary calcium is not a precise marker of bone resorption. Deoxypyridinoline seems to be the most specific bone resorption marker in cancer patients. Biochemical responders have the most benefit from pamidronate in terms of pain relief. This suggests that patients may benefit from more potent or repeated infusions of bisphosphonates.

**Keywords:** pamidronate; bone metastasis; pyridinium cross-link; bisphosphonate; bone resorption

Bone is the most common site for metastases from breast and prostate cancer, affecting about 70% of patients with metastatic disease. These metastases cause considerable morbidity including bone pain, hypercalcaemia, pathological fractures, nerve root/spinal cord compression and consequently a decrease in mobility and quality of life. Palliation of symptoms and improvement in quality of life are the major therapeutic goals in metastatic bone disease.

Our understanding of the pathophysiology of bone metastases has increased in the last few years. The most important mechanism of bone destruction is the release of paracrine factors (cytokines, growth factors, prostaglandins), which stimulate osteoclasts to resorb bone (Boyce, 1991). There is some recent evidence that bone is itself an important source of growth factors, which following release during bone resorption, may stimulate tumour growth (Garret, 1993), creating a stimulatory vicious circle in the bone microenvironment.

Bisphosphonates are agents that inhibit osteoclastic bone resorption in several ways and might be able to disrupt this cycle. They may have a direct cytotoxic effect on osteoclasts, inhibit the migration and transformation of osteoclast precursors into mature osteoclasts and impede the attachment of osteoclasts to the bone surface (Flanagan and Chambers, 1989; Lowik *et al.*, 1988).

Bisphosphonates are increasingly being prescribed for patients with bone metastases. They are the treatment of choice in hypercalcaemia of malignancy and may be used in the management of bone pain from bone metastases. They are also able to prevent skeletal complications such as pain requiring radiotherapy, reduce the incidence of hypercalcaemia and pathological fractures (Van Holten-Verzantvoort *et al.*, 1993; Paterson *et al.*, 1993; Lahtinen *et al.*, 1992) and, when associated with chemotherapy, increase the median time to disease progression in bone (Conte *et al.*, 1994).

Correspondence: J Vinholes
Received 6 September 1995; revised 28 November 1995; accepted 29 November 1995

There is uncertainty about the ideal schedule of pamidronate in metastatic bone disease because of the difficulty in monitoring its effects. Measurement of bone turnover by biochemical markers is perhaps a more objective method to evaluate the effects of bisphosphonates on bone. Urinary calcium (uCa) has traditionally been used to evaluate bone resorption (Campbell *et al.*, 1983) and is considered the standard bone resorption marker in oncology, although it does not exclusively reflect bone resorption. Hydroxyproline (Hyp) is another traditional bone resorption marker that has been used to evaluate pharmacological effects on bone turnover, but has its own sources of inaccuracy (Coleman *et al.*, 1988).

There is a clear need for more reliable and specific markers of bone resorption. In the last few years, new products of collagen breakdown, which are released into the circulation during bone resorption, have been identified as bone resorption markers. They are the naturally fluorescent non-reducible cross-links of collagen: deoxypyridinoline and pyridinoline (Eyre *et al.*, 1984). Their function is to stabilise the collagen fibrils by forming bridge linkages (cross-links) with neighbouring collagen molecules (Eastell, 1994). Pyridinoline is also found in cartilage, ligament, tendons, vessels and can be released from the tumour stroma whereas deoxypyridinoline is found in significant amounts only in bone and therefore is highly bone-specific (Eyre, 1992). Cross-links are not influenced by diet (Colwell *et al.*, 1993), probably not metabolised, and excreted in the urine. They have primarily been tested in Paget's disease of bone and osteoporosis. Oncology is a new area of application for these markers. We have previously reported preliminary results on the use of urinary excretion of pyridinium cross-links for monitoring metastatic bone disease (Coleman *et al.*, 1992).

Pamidronate is the most potent bisphosphonate in clinical use. Our group has previously reported the clinical results of this trial of high-dose pamidronate elsewhere (Purohit *et al.*, 1994). The aims of this study were to confirm the value of specific resorption markers in monitoring metastatic bone disease and define the duration of action of a single pamidronate infusion.

### Patients and methods

### *Patients*

We were able to perform detailed biochemical analysis in 20 of 34 patients that were recruited to this clinical trial. These patients were only chosen because they had a complete set of samples collected over 8 weeks after the infusion. The percentage of patients with pain relief in this subgroup was similar to the percentage of the whole group. Five were perimenopausal and 12 post-menopausal women (mean age 48 years, range 32–64; mean years post-menopausal 3, range 0–10) and 3 were men (mean age 63 years, range 51–75). Fifteen patients had breast cancer, three prostate cancer and two others. Because of other ongoing trials involving patients with lytic bone metastases most patients had predominantly sclerotic bone metastases.

Entry criteria included patients with painful radiologically confirmed bone metastases, at least one previous chemo- or hormonal therapy, no bisphosphonate treatment in the previous 3 months, no other drugs affecting bone metabolism and no concomitant systemic treatment. Analgesics were allowed. All patients were normocalcaemic at entry to the study.

The control group consisted of 20 healthy volunteers, 17 women (mean age 51 years, range 47–59; mean years post-menopausal 4, range 0–10) matched for years post-menopausal and three men (mean age 59, range 48–71) matched for age. Fasting serum samples and a second voided urine sample were collected from them.

All patients received an infusion of pamidronate 120 mg and were followed up over 8 weeks. Ten patients subsequently received a second infusion with the same dose for recurrence of bone pain. This second infusion was given with a median of 6 weeks (range 2–10) after the 8 weeks follow-up of the first treatment. Pamidronate was given over 12 hours in 1 l normal saline and subjects were followed up in the out-patient department.

### *Biochemical analysis*

A fasting morning serum sample and a second voided urine sample were collected at baseline, 2, 4 and 8 weeks after both treatments and stored at −20°C. The urine was acidified with 3% hydrochloric acid before storage. All samples from an individual were analysed in duplicate and in the same batch to minimise interassay variation.

Routine serum measurements included full blood count (FBC), serum calcium (sCa) corrected for albumin, phosphate ($PO_4$), creatinine, urea, parathyroid hormone (PTH) and total alkaline phosphatase. Two bone formation markers were also measured: osteocalcin (Oc) and bone alkaline phosphatase (bAP).

Intact PTH was measured by a two-site immunoradiometric assay (Allegro PTH, Nichols Institute, San Juan Capistrano, CA, USA). The detection limit of the assay is 1 pg $ml^{-1}$. The intra-assay and interassay variability were 5% and 5.5% respectively. Osteocalcin was measured by a two-site immunoradiometric assay that measures intact osteocalcin (1–49 peptides) and the large N-terminal mid-fragment (1–43 peptides) using human osteocalcin as standard (Elsa-Osteo, CIS, Gif-sur-Yvette, France). Samples may be stored at −20°C for this osteocalcin assay as it is less sensitive to storage conditions than other commercial assays (Blumsohn, 1995*a*). This assay is claimed to have less *in vitro* proteolytic degradation when exposed to room temperature than other assays used to measure osteocalcin (Garnero *et al*., 1994*a*). The detection limit of the assay is 0.4 ng $ml^{-1}$. The intra-assay and interassay variability were 5.8% and 7% respectively. Bone alkaline phosphatase was measured by a precipitation method that makes use of the affinity of the wheatgerm lectin for the bone isoenzyme (Rosalki and Foo, 1984). The amount of the bone isoenzyme is calculated by subtracting the total alkaline phosphatase from the supernatant (liver isoenzyme). Roche uni-kit III was used for analysis with the application of a COBAS machine. The intra-assay and interassay variability were 5.8% and 7% respectively.

Urinary creatinine was measured using an automated chemistry analyser using a kinetic Jaffe method and all the urinary markers are expressed as a ratio to creatinine excretion. Urinary calcium was measured by a colorimetric assay. Hydroxyproline was measured by a colorimetric assay with dimethylaminobenzaldehyde after acid hydrolysis and chloramine T oxidation.

Total pyridinoline (Pyd) and deoxypyridinoline (Dpd) were measured in duplicate after acid hydrolysis, CF1 cellulose column partition chromatography, and reverse-phase high-performance liquid chromatography (HPLC) with fluorescence detection (Colwell *et al*., 1993). The intra-assay and interassay variability were 8.5% and 12.5% respectively.

### *Response criteria*

At each visit the scores for pain intensity, WHO performance status and analgesic consumption were combined to produce an overall pain score (Coleman, 1994). Subjective response to treatment was defined as ⩾20% decrease in the pain score compared with the baseline on at least two consecutive measurements. Patients with pain decrease <20% were considered non-subjective responders (Purohit *et al*., 1994). Biochemical response to treatment was defined as ⩾50% decrease in the deoxypyridinoline (which is the most bone-specific resorption marker) value compared with the baseline value. Patients with <50% decrease in the deoxypyridinoline values were considered as non-biochemical responders. This value was chosen because this is the usual cut-off point between response or not to treatment in cancer patients.

### *Statistical methods*

Most markers showed a skewed distribution, so the data were logarithmically and back transformed, expressed as mean ± standard error and shown as the percentage (%) of baseline values of each infusion, except for serum calcium and phosphate which are expressed as mmol $l^{-1}$. For comparison of baseline measurements of patients and controls, the unpaired *t*-test was used. The reference range was defined non-parametrically as between the 2.5% and 97.5% percentiles. A paired *t*-test with Bonferroni correction was used to compare changes in markers over time. As three comparisons were made, $P<0.017$ was considered significant. Baseline values of both infusions were compared by the paired *t*-test with $P<0.05$ considered as significant. The area under the response curve (AUC) of each marker in both infusions was determined and compared with an AUC assuming no change over the 8 weeks with $P<0.05$ considered as significant.

Comparisons between biochemical responders and non-responders and between first and second infusions were done by multifactor analysis of variance using the Scheffe test. Pearson correlation coefficients between markers at baseline and between AUC after treatment were determined. Pearson correlation was also used to compare percentage of pain score change and percentage decrease in deoxypyridinoline levels.

### Results

### *General biochemistry and haematology*

Serum calcium showed a significant decrease after the first infusion at 2 and 4 weeks (Table I). The AUC for calcium after the first infusion also showed a significant decrease ($P<0.003$). Serum calcium also fell after the second infusion, but this was not significant. The AUC of serum phosphate fell significantly after both infusions ($P<0.02$ after the first infusion and $P<0.01$ after the second infusion). PTH levels increased at 2 and 4 weeks after the first infusion by >100%, (Table I), with every patient showing at least a 20% increase compared with baseline values. The AUC also showed a significant increase ($P<0.005$). Following the second infusion, PTH increased by 65% at 2 weeks ($P<0.01$) and by 23% at 4 weeks (NS) (Table I).

*Bone formation*

Baseline values of bone and total alkaline phosphatase were increased significantly, contrasting with osteocalcin results (Table II). Osteocalcin and bone alkaline phosphatase decreased by 15% at 8 weeks after both infusions, but this was not statistically significant (Figure 1). Bone alkaline phosphatase decreased by 30% at 8 weeks after the second infusion compared with the baseline of the first infusion, but this also did not reach statistical significance. There was no correlation between osteocalcin and bone alkaline phosphatase at baseline ($r=0.3$) or AUC of the first infusion ($r=0.2$).

*Bone resorption*

All resorption markers, except urinary calcium, were increased significantly at the beginning of treatment, with pyridinoline and deoxypyridinoline being the most frequently increased markers (70%). The spread of pretreatment values is shown in Figure 2. Before the second infusion, only pyridinoline and deoxypyridinoline were significantly increased when compared with controls ($t$-test). Urinary calcium was increased in only 40% and 20% of patients before the first and second infusions respectively (Table II).

**Figure 1** Bone formation markers after the first infusion. Values expressed as percentage of the mean±s.e.m. (no significant changes). bAP, (- - -); Oc (—).

Urinary calcium, hydroxyproline, pyridinoline and deoxypyridinoline fell significantly at all time points after the first infusion (Figure 3). The AUC approach also showed that there was a significant decrease in these markers ($P<0.001$). After the second infusion, the resorption markers fell significantly at 2 and 4 weeks compared with baseline of this infusion except for hydroxyproline, owing to high interpatient variability. The shape of the curve response of this infusion was similar to the first infusion.

Before the first treatment, 70% patients had increased Dpd values. Within a month, 50% of these patients had normalised Dpd values. However, after 8 weeks only 15% of these patients still had normal values. Before the second infusion, 70% patients had increased Dpd values. Within a month, >50% of patients had normalised Dpd values.

**Figure 2** Baseline values of resorption markers. The upper limit of normal for each marker is indicated by a line.

**Table I** Serum calcium, phosphate and PTH levels expressed as mean ± s.e.m.

| | First infusion | | | | Second infusion | | | |
|---|---|---|---|---|---|---|---|---|
| | *Baseline* | *2 weeks* | *4 weeks* | *8 weeks* | *Baseline* | *2 weeks* | *4 weeks* | *8 weeks* |
| Ca | 2.4±.03 | 2.2±.03* | 2.3±.03* | 2.3±.03 | 2.3±.05 | 2.2±.05 | 2.3±.05 | 2.3±.05 |
| $PO_4$ | 1.2±.04 | 1.0±.04* | 1.1±.05 | 1.1±.05 | 1.23±.07 | 1.09±.07 | 1.08±.07* | 1.1±.06 |
| PTH | 100 | 220±29* | 215±31* | 127±21 | 100 | 162±19* | 123±11 | 118±18 |

Ca and $PO_4$ expressed as mmol $l^{-1}$. PTH expressed as percentage of baseline levels. Paired $t$-test with Bonferroni correction. *$P<0.017$.

**Table II** Baseline markers of bone turnover measured in controls and patients at first and second infusions

| | *Oc* ng ml⁻¹ | *Bap* U l⁻¹ | *Hyp* μmol mmol⁻¹ | *Uca* mmol mmol⁻¹ | *Dpd* nmol mmol⁻¹ | *Pyd* nmol mmol⁻¹ |
|---|---|---|---|---|---|---|
| Control mean | 22 | 35 | 22 | 0.27 | 6.1 | 24 |
| 2.5–97.5 percentiles | 12–36.6 | 25–65.3 | 12.7–41.6 | 0.09–0.42 | 3.4–11.8 | 15.2–41.7 |
| Patients mean ± s.e.m. – first infusion | 25±5 | 67±15** | 47±6** | 0.35±0.06 | 15±2.5** | 62±12** |
| Percentage of patients with high values – first infusion | 40% | 65% | 55% | 40% | 70% | 70% |
| Patients mean ± s.e.m. – second infusion | 32±15 | 65±18* | 32±9 | 0.24±0.06 | 14±5* | 63±16** |
| Percentage of patients with high values – second infusion | 40% | 50% | 50% | 20% | 70% | 70% |

Urinary markers are expressed as a ratio to creatinine. *$P<0.05$, **$P<0.01$, in comparison with controls.

However, after 8 weeks only 20% of patients still had normal values. Before the first treatment, 40% had increased uCa levels. Within a month, 90% of patients had normal levels, which persisted for >8 weeks. Before the first infusion, 70% and 55% of patients had increased Pyd and Hyp values respectively. Among them, 30% and 50% of patients normalised these values respectively.

The Pyd/Dpd ratio was significantly higher in patients at baseline compared with controls (4.4 *vs* 3.9 respectively). This ratio increased significantly at 2 weeks to 6.5 and then decreased to 5.7 at 4 weeks and 5.1 at 8 weeks. The effects of each treatment on bone turnover markers over the 8 weeks were compared by multifactor analysis of variance. No significant differences in biochemical effects between the two infusions was identified ($P=0.22$). Urinary calcium was excluded from this analysis because its levels were influenced by PTH.

### *Pain relief and biochemical response*

Two patients received dexamethasone and radiotherapy, which make the interpretation of the change in the pain score difficult, were considered non-assessable. The mean pain score in the remainder fell at 2 weeks and remained practically unchanged to 8 weeks (Figure 4), reflecting the decrease in bone resorption. Before the second infusion, the pain score increased again but fell with retreatment (Figure 4).

Thirteen patients showed a subjective response. Of these, 11 were also biochemical responders. The other two patients only had a 30% reduction in Dpd. Five patients were non-subjective responders and of these, only two were considered biochemical responders.

In total, there were 13 biochemical responders and seven non-responders. All of the non-responders also showed <50% reduction in pyridinoline and four had <50% reduction in hydroxyproline values, whereas all of them showed ⩾50% reduction in urinary calcium levels.

Biochemical responders showed a greater relief of pain when compared with non-responders by multifactor analysis of variance ($P<0.01$) (Figure 5). There was a significant correlation between the maximum decrease in pain score (%) and the maximum decrease (%) in deoxypyridinoline levels ($r=0.51$, $P<0.05$). However, changes in pain score did not correlate with changes in any of the other resorption markers.

Seven out of ten patients responded subjectively to treatment after the second infusion. Among these, six were biochemical responders. Three patients did not show pain relief. Among those, two were considered non-biochemical responders.

In the ten patients who received both infusions, baseline levels of bone markers at both infusions were compared. Although there was some carry-over effect from the first infusion (Figure 4), none of the markers showed a statistically significant difference. Pearson correlation coefficients of the baseline and AUC values after the first infusion are shown in Table III. There was a significant correlation between Dpd, Pyd and Hyp before and during treatment (AUC values). However there was no correlation at any time with urinary calcium. After the second infusion, correlations between Dpd, Pyd and Hyp were significant at baseline: Dpd

**Figure 4** Pain score and bone resorption markers of the ten patients that received two infusions. Resorption markers expressed as percentage of the mean of pretreatment values.— - - —, Pain; – · – · -, Pyd;- - - - -, Hyp; ———, Dpd; – – – , uCa.

**Figure 3** Bone resorption markers after the first infusion. Values expressed as percentage of the mean±s.e.m. ($P<0.017$ at all time points). -○-, Pyd; - - -◇- - -, Hyp; -△-, Dpd; -□-, uCa.

**Figure 5** Pain score comparison of objective and non-objective responders after first infusion. Significance level by multifactor analysis of variance. ($P=<0.01$). - - -, Non-biochemical responders; ——, biochemical responders.

**Table III** Pearson correlation coefficients of markers at first infusion

| | *uCa* | | *Hyp* | | *Pyd* | | *Dpd* | |
|---|---|---|---|---|---|---|---|---|
| | *Baseline* | *AUC* | *Baseline* | *AUC* | *Baseline* | *AUC* | *Baseline* | *AUC* |
| uCa | – | – | 0.15 | 0.27 | 0.17 | –0.06 | 0.19 | –0.05 |
| Hyp | 0.1 | 0.3 | – | – | 0.83** | 0.5* | 0.74** | 0.5* |
| Pyd | 0.17 | –0.06 | 0.83** | 0.5* | – | – | 0.9** | 0.88** |
| Dpd | 0.19 | –0.05 | 0.74** | 0.5* | 0.9** | 0.88** | – | – |

AUC, area under the curve. *$P<0.05$, **$P<0.003$.

*vs* Pyd: $r=0.92$, $P<0.005$; Dpd *vs* Hyp: $r=0.88$, $P<0.017$; Pyd *vs* Hyp: $r=0.77$, $P<0.017$, but none of them correlated with uCa. The AUC of Dpd and Pyd correlated significantly ($r=0.72$, $P<0.05$), but again none of them correlated with urinary calcium.

### Discussion

Objective evaluation of the response in bone to therapy is a challenge. Therefore, there is a clear need for new methods of response assessment in bone. Changes in biochemical markers can be determined in the first few weeks after the beginning of a therapy. There are a number of markers being used in oncology to monitor cancer therapy, such as CA-125, α-fetoprotein and human chorionic gonadothrophin.

In this trial, pamidronate induced a profound fall in bone resorption, with a maximum effect seen at 2 weeks. Indeed, studies in hypercalcaemia of malignancy (Coleman and Rubens, 1987; Vinholes *et al.*, 1995) suggest that the maximal effect on bone resorption occurs even earlier than 2 weeks. After two weeks there was a steady progressive increase in bone resorption, but significant inhibition persisted throughout the 8 weeks of observation.

The inhibition of bone resorption reduces the release of calcium from the skeleton to the circulation, which was evidenced by the fall in serum calcium after both treatments. A statistically significant fall in the serum calcium of normocalcaemic breast or prostate cancer patients has previously been observed after clodronate (Thiebaud *et al.*, 1991; Martoni *et al.*, 1991) and pamidronate (Lipton *et al.*, 1994). As calcium homeostasis is tightly controlled by PTH, even relatively small falls in calcium levels, induce a negative feedback in the parathyroid glands within a few minutes. The accuracy of this process is impressive, allowing serum calcium to change only about 0.025 mmol $l^{-1}$ during the day (Broadus, 1993). We observed a marked increase in PTH levels after both infusions, with some effects still persisting at 8 weeks. A significant increase in PTH levels after oral and intravenous (i.v.) pamidronate was also found by other investigators (Reid *et al.*, 1994; Body *et al.*, 1995) and also after i.v. clodronate (Pecherstorfer *et al.*, 1993). PTH acutely regulates serum calcium by increasing distal renal tubular reabsorption of calcium, hence decreasing urinary calcium excretion. Furthermore, PTH leads to a decrease in distal and proximal renal tubular reabsorption of phosphate, resetting the renal tubular threshold of phosphate at a lower level.

Urinary calcium has been used to measure bone resorption for more than three decades. Most published reports about the effects of bisphosphonates in cancer patients have relied on urinary calcium to monitor bone resorption (O'Rourke *et al.*, 1995). Although uCa is a cheap and easy to measure assay, it can be influenced by diet, renal function, parathyroid hormone, parathyroid hormone-related protein and seems to reflect the balance of bone turnover, rather than bone resorption specifically (Blomqvist *et al.*, 1987). Compared with the other resorption markers, relatively few patients had increased baseline values in this trial. This confirms the findings of a recently published study that compared 143 normal controls with 98 cancer patients with bone metastases and showed no significant increase in urinary calcium between cancer patients (mainly breast cancer) with bone metastases and controls (Pecherstorfer *et al.*, 1995). In addition in our study, within a month after pamidronate, <10% of patients had abnormal urinary calcium values, while 35% of patients had Dpd levels above the normal range. Therefore, urinary calcium does not seem to be a sensitive marker of bone resorption.

Not surprisingly, urinary calcium did not correlate with any other resorption marker at baseline or by comparison of areas under the curve after both treatments. This lack of correlation with other resorption markers has also been noted in previous studies (Coleman *et al.*, 1992; Body and Delmas, 1992). This is explained by the fact that urinary calcium is not only monitoring changes in bone turnover, but is also reflecting the renal handling of calcium by PTH whereas cross-links reflect the metabolism of the bone collagen (Seibel *et al.*, 1994). The absence of correlation of changes in urinary calcium with pain score confirms some recently published data (O'Rourke *et al.*, 1995).

Hydroxyproline is an imino acid present in significant amount in all collagenous tissues (including bone), elastin and complement factor Clq. It is estimated that about 80% of hydroxyproline is metabolised in the liver by hydroxyproline oxidase and only 10% is excreted in the urine (Eastell, 1994). Because serum levels of hydroxyproline can be influenced by diet or soft tissue destruction by extraskeletal metastases it does not have the profile of an accurate resorption marker. The fall in hydroxyproline was not statistically significant after the second infusion.

Pyridinoline and deoxypyridinoline were increased in 70% of patients. They have also been shown to be significantly increased in other reports (Coleman *et al.*, 1992; Lipton *et al.*, 1993; Pecherstorfer *et al.*, 1995). Ninety per cent of patients had mainly osteosclerotic disease, in which bone resorption is not increased as much as it is in lytic or mixed bone metastases.

Pyridinoline and deoxypyridinoline showed a consistent mean decrease of 30% and 42% respectively after both infusions. There was also a significant correlation between Pyd and Dpd at baseline and with treatment using the AUC values, with hydroxyproline, but no correlation with urinary calcium.

The Dpd/Pyd ratio was increased in these patients, which is supported by recent similar findings (Pecherstorfer *et al.*, 1995; Behrens *et al.*, 1995). This increase may be explained by the fact that many patients also had concomitant soft tissue metastases and it is known that there is a soft tissue component to the urinary excretion of pyridinoline. These soft tissue metastases possibly progressed during the trial as these patients were not receiving any systemic anti-cancer therapy. The decrease in the ratio towards normal after 2 weeks is probably due to the reactivating bone metastases. Dpd showed a greater decrease than Pyd after both infusions, resulting in a significant increase in the Pyd/Dpd ratio after treatment, probably reflecting the higher bone specificity of deoxypyridinoline. Similar observations were made in a previous study on oral pamidronate (Coleman *et al.*, 1992). This increased Pyd/Dpd ratio in cancer patients suggests that deoxypyridinoline is a more reliable marker in this situation than pyridinoline.

The changes in pain score seem to follow the changes in resorption markers and suggest that these markers could be used to monitor antiresorptive therapy. At the time of the second treatment when patients had recurrent bone pain, the pamidronate effects on bone resorption had faded away and bone resorption was increasing again. This suggests a positive relationship between increased bone resorption and bone pain and the need of further treatment in responsive patients. The significant, although somewhat weak correlation, between changes in Dpd and pain score also reinforces this possibility. The significantly greater decrease in pain score in patients considered as biochemical responders is interesting and suggests that more potent bisphosphonates that are capable of reducing bone resorption further may be clinically superior to currently available compounds. We have taken 50% as a cut-off point because this is a widely accepted index of markers and biochemical response in clinical oncology and we intend to evaluate this prospectively in a trial in which we are comparing high-dose pamidronate with placebo for painful bone metastases.

Bone formation markers remained unchanged over the first month, beginning to fall at 8 weeks and with some carry-over effect to the next dose. Close follow-up over a longer period of time is needed to assess possible clinically important reductions in the rate of bone formation with long-term bisphosphonate use. Bone alkaline phosphatase was elevated in two-thirds of the patients and was a more sensitive indicator of bone formation than osteocalcin, which was elevated in only 40% of patients at baseline. Interestingly, there was no significant correlation between these formation markers, as previously described (Li et al., 1993), possibly because bone alkaline phosphatase reflects the synthesis of the organic bone matrix while osteocalcin reflects the mineralisation process.

The shape of the time curve response of bone metabolism markers was similar after both infusions. Resorption markers are the first to decrease and are followed by the formation markers about 6–8 weeks later. This reflects the coupling process of bone resorption and formation, in a process known as bone remodelling. The entire process takes about 3–4 months with bone formation only normally occurring in areas of bone undergoing resorption (Mundy, 1995).

The optimal choice of bone metabolism markers remains an area for debate. Bone formation markers only begin to fall about 8 weeks after therapy, so they obviously cannot reflect the effects of therapy before this time. Among the resorption markers, deoxypyridinoline seems to be the best candidate to monitor therapy. Patients with abnormal Dpd values might benefit from a second infusion of pamidronate if these values are still above the normal range and/or to perpetuate a subjective response.

Although deoxypyridinoline is a more specific and reliable resorption marker, the HPLC technique is notoriously cumbersome and time consuming, an important obstacle to routine use in monitoring therapy in cancer patients. Recently, some immunoassays to measure peptide-bound cross-links such as the N-telopeptide–NTX–(Hanson et al., 1992) and the C-telopeptide of type I collagen–Crosslaps–(Garnero, 1994b) have been developed. They have shown a good correlation with deoxypyridinoline and pyridinoline and are much easier to measure (Blumsohn et al., 1995b).

In conclusion, we can confirm that pamidronate is a potent inhibitor of bone resorption in patients with metastatic bone disease. Comparative trials of dose, potency and schedule are needed to determine the true relationship between biochemical and clinical effects, but our paper suggests that repeated doses or more potent bisphosphonates may be clinically even more effective. As there is a possibility that a placebo effect could have contributed to the subjective response, we are now carrying out a randomised double-blind placebo-controlled study. Urinary calcium is not a sensitive or specific marker of bone resorption. Deoxypyridinoline seems to be the best resorption marker to monitor therapy, although pain is complex and only partially related to the rate of bone resorption. The availability of specific bone resorption markers will be helpful for selecting and monitoring the optimal schedule of bisphosphonates in cancer patients.

**Acknowledgements**
J Vinholes was supported by a grant from the European Organisation for Research and Treatment of Cancer–Breast Cancer Cooperative Group.

### References

BEHRENS P, KUIRAOKA M, MULLER PK AND ACYL Y. (1995). Pyridinium cross-links as a resorption marker in the diagnosis for osteopathy. *Calcif. Tissue Int.*, **56**, 446.

BLOMQVIST C, ELOMAA I, RISTELI J, VIRKKUNEN P, PORKKA L, KARONEN SL, RISTELI L AND RISTELI J. (1987). The response evaluation of bone metastases in mammary carcinoma. The value of radiology, scintigraphy and biochemical markers of bone metabolism. *Cancer*, **60**, 2907–2912.

BLUMSOHN A, HANNON R AND EASTELL R. (1995*a*). Apparent instability of osteocalcin in serum as measured with different commercially available immunoassays. *Clin. Chem.*, **41**, 318–319.

BLUMSOHN A, NAYLOR KE, ASSIRI AMA AND EASTELL R. (1995*b*). Different responses of biochemical markers of bone resorption to bisphosphonate therapy in Paget's disease. *Clin. Chem.* **41**, 1592–1598.

BODY JJ AND DELMAS PD. (1992). Urinary pyridinium cross-links as markers of bone resorption in tumour-associated hypercalcaemia. *J. Clin. Endocrinol. Metab.*, **74**, 471–475.

BODY JJ, DUMON JC, MENDES M AND LUMEN AA. (1995). Biochemical dose–response effects of pamidronate in postmenopausal osteoporosis–evaluation of new markers of bone resorption. *Calcif. Tissue Int.*, **56**, 481.

BOYCE BF. (1991). Normal bone remodelling and its disruption in metastatic bone disease. In *Bone Metastases–Diagnosis and Treatment*, Rubens RD and Fogelman I (eds), pp. 11–30, Springer: London.

BROADUS AE. (1993). Physiological functions of calcium, magnesium, phosphorus and mineral ion balance. In *Primers on the Metabolic Bone Diseases and Disorders of Mineral Metabolism*, Favus MJ (ed), pp. 41–46, Raven Press: New York.

CAMPBELL FC, BLAMEY RW, WOOLFSON AMJ, ELSTON CW AND HOSKING DT. (1983). Calcium excretion in metastatic breast cancer. *Br. J. Surg.*, **70**, 202.

COLEMAN RE. (1994). Evaluation of bone disease in breast cancer. *Breast*, **3**, 73–78.

COLEMAN RE AND RUBENS R. (1987). Treatment of hypercalcaemia of malignancy secondary to advanced breast cancer with pamidronate. *Br. J. Cancer*, **56**, 465–469.

COLEMAN RE, WHITAKER KD, MOSS DW, MASHITER G, FOGELMAN I AND RUBENS RD. (1988). Biochemical monitoring predicts response in bone to systemic treatment. *Br. J. Cancer*, **58**, 205–210.

COLEMAN RE, HOUSTON S, JAMES I, RODGER A, RUBENS RD, LEONARD RCF AND FORD J. (1992). Preliminary results of the use of urinary excretion of pyridinium cross-links for monitoring metastatic bone disease. *Br. J. Cancer*, **65**, 766–768.

COLWELL A, RUSSELL RGG AND EASTELL R. (1993). Factors affecting the assay of urinary 3-hydroxy pyridinium cross-links of collagen as markers of bone resorption. *Eur. J. Clin Invest.*, **23**, 341–349.

CONTE PF, GIANNESSI PG, LATREILLE J, MAURIAC L, KOLIREN L, CLABRESI F AND FORD JM. (1994). Delayed progression of bone metastases with pamidronate therapy in breast cancer patients: a randomised, multicenter phase III trial. *Ann. Oncol.*, **5** (suppl. 7), S41–44.

EASTELL R. (1994). Biochemical markers. In *Spine–State of the Art Reviews*, 8: 1, pp. 155–170, Hanley & Belfus: Philadelphia.

EYRE DR. (1992). New biomarkers of bone resorption. *J. Clin. Endocrinol. Metab.*, **74**, 470.

EYRE DR, KOOB TJ AND VAN NESS KP. (1984). Quantification of hydroxypyridinium cross-links in collagen by high performance liquid chromatography. *Ann. Biochem.*, **137**, 380–388.

FLANAGAN AM AND CHAMBERS TJ. (1989). Clodronate inhibits resorption through injury of osteoclast that resorbs clodronate coated bone. *Bone Miner.*, **6**, 33–43.

GARNERO P, GRIMAUX M, SEGUIN P AND DELMAS P. (1994*a*). Characterisation of immunoreactive forms of human osteocalcin generated in vivo and in vitro. *J. Bone. Miner. Res.*, **9**, 255–264.

GARNERO P, GINEYTS E, RIOU JP AND DELMAS PD. (1994*b*). Assessment of bone resorption with a new marker of collagen degradation in patients with metabolic bone disease. *J. Clin. Endocrinol. Metab.*, **79**, 780–785.

GARRET R. (1993). Bone destruction in cancer. *Semin. Oncol.*, **20** (suppl. 2), 4–9.

HANSON DA, WEIS MAE, BOLLEN AM, MASLAN SL, SINGER FR AND EYRE DR. (1992). A specific immunoassay for monitoring human bone resorption: quantification of type I collagen cross-linked N-telopeptides in urine. *J. Bone Miner. Res.*, **7**, 1251–1258.

LAHTINEN R, LAASKO M, PALVA I, VIRKKUEN P, AND ELOMAA I FOR THE FINNISH LEUKAEMIA GROUP. (1992). Randomised, placebo-controlled multicentre trial of clodronate in multiple myeloma. *Lancet*, **340**, 1049.

LI F, PITT PI, SHERWOOD R, BARRETT J, HOUGHTON J, PARSONS V AND MONIZ C. (1993). Biochemical markers of bone turnover in women with surgically treated carcinoma of the breast. *Eur. J. Clin. Invest.*, **23**, 566–571.

LIPTON A, DEMERS L, DANILOFF Y, CURLEY E, HAMILTON C, HARVEY H, WITTERS L, SEAMAN J. VAN DER GIESSEN R AND SEYEDIN S. (1993). Increased urinary excretion of pyridinium cross-links in cancer patients. *Clin. Chem.*, **39**, 614–618.

LIPTON A, GLOVER D, HARVEY H, GRABELSKY S, ZELENAKAS K, MACERATA R AND SEAMAN J. (1994). Pamidronate in the treatment of bone metastases: results of 2 dose-ranging trials in patients with breast or prostate cancer. *Ann. Oncol.*, **5** (suppl. 7), 31–35.

LOWIK CWGM, VAN DER PLUIJM G AND BIJVOET OLM. (1988). Migration and phenotypic transformation of osteoclast precursors into mature osteoclasts, the effects of a bisphosphonate. *J. Bone Miner. Res.*, **3,** 185–192.

MARTONI A, GUARALDI M, CAMERA P, BIAGI R, MARRI S, BEGHE F AND PANNUTI F. (1991). Controlled clinical study on the use of dichloromethylene disphosphonate in patients with breast carcinoma metastasizing to the skeleton. *Oncol.*, **48,** 97–101.

MUNDY G. (1995). Metastatic bone disease. In *Bone Remodelling and its Disorders*, Mundy G (ed), pp. 104–122, Martin Dunitz: London.

O'ROURKE N, MCCLOSKEY E, HOUGHTON F, HUSS H AND KANIS JA. (1995). Double-blind, placebo-controlled, dose-response trial of oral clodronate in patients with bone metastases. *J. Clin. Oncol.*, **13,** 929–934.

PATERSON AHG, POWLES TJ, KANIS JA, MCCLOSKEY E, HANSON J AND ASHLEY S. (1993). Double-blind controlled trial of oral clodronate in patients with bone metastases from breast cancer. *J. Clin. Oncol.*, **11,** 59–65.

PECHERSTORFER M, SCHILING T, JANISCH S, WOLOSZCZUK W, BAUMGARTNER G, ZIEGLER R AND OGRIS E. (1993). Effect of clodronate treatment on bone scintigraphy in metastatic breast cancer. *J. Nucl. Med.*, **34,** 1039–1044.

PECHERSTORFER M, LUDWIG H, ZIMMER-ROTH, SCHILING T, WOITGE HW, SCHIMIDT H, BAUMGARTNER G, THIEBAUD D AND SEIBEL M. (1995). The diagnostic value of urinary pyridinium cross-links of collagen, alkaline phosphatase and urinary calcium excretion in neoplastic bone disease. *J. Clin. Endocrinol. Metab.*, **80,** 97–103.

PUROHIT OP, ANTHONY C, OWEN J, RADSTONE CR AND COLEMAN RE. (1994). High-dose intravenous pamidronate for metastatic bone pain. *Br. J. Cancer*, **70,** 554–558.

REID IR, WATTIE DJ, EVANS MC, GAMBLE GD, STAPLETON JP AND CORNISH J. (1994). Continuous therapy with pamidronate, a potent bisphosphonate, in postmenopausal osteoporosis. *J. Clin. Endocrinol. Metab.*, **79,** 1595–1599.

ROSALKI SB AND FOO AY. (1984). Two new methods for separating and quantifying bone and liver alkaline phosphatase isoenzymes in plasma. *Clin. Chem.*, **30,** 1182–1186.

SEIBEL MJ, LAMBRINOUDARI IL AND ZIPF A. (1994). Biochemical markers of bone metabolism in metastatic bone disease. In *Metastatic Bone Disease*, Diel J, Kaufmann M and Bastert G. (eds), pp. 108–125. Springer-Verlag: Berlin.

THIEBAUD D, LEYVRAZ S, VON FLIEDNER V, PERCY L AND BURCKHARDT P. (1991). Treatment of bone metastases from breast cancer and myeloma with pamidronate. *Eur. J. Cancer*, **27,** pp. 37–41.

VAN HOLTEN-VERZANTVOORT ATM, KROON HM, BIJVOET OLM, CLETON FJ, BEEX LVAM, BLIJHAM G, HERMANS J, NEIJT JP, PAPAPOULOS SE, SLEEBOOM HP, VERMEY P AND ZWINDERMAN AH. (1993). Palliative pamidronate treatment in patients with bone metastases from breast cancer. *J. Clin. Oncol.*, **11,** 491–498.

VINHOLES J, PUROHIT OP, GUO CY, EASTELL R AND COLEMAN R. (1995). Randomised double-blind comparison of pamidronate or clodronate for hypercalcaemia of malignancy: effects on bone metabolism markers. *Eur. J. Cancer*, **31A** (suppl. 5), S253.